# Polymer-Based Honeycomb Films on Bioactive Glass: Toward a Biphasic Material for Bone Tissue Engineering Applications

**DOI:** 10.1021/acsami.1c03759

**Published:** 2021-06-15

**Authors:** A. Deraine, M. T. Rebelo Calejo, R. Agniel, M. Kellomäki, E. Pauthe, M. Boissière, J. Massera

**Affiliations:** † ERRMECe, Equipe de Recherche sur les Relations Matrice Extracellulaire-Cellules (EA1391), Université de Cergy-Pontoise, Maison Internationale de la Recherche (MIR), Rue Descartes, 95001 Neuville sur Oise, Cedex, France; ‡ Laboratory of Biomaterials and Tissue Engineering, Faculty of Medicine and Health Technology, Tampere University, Korkeakoulunkatu 3, 33720 Tampere, Finland

**Keywords:** bioactive glass, honeycomb membrane, biphasic material, bone tissue engineering, *in vitro* stability

## Abstract

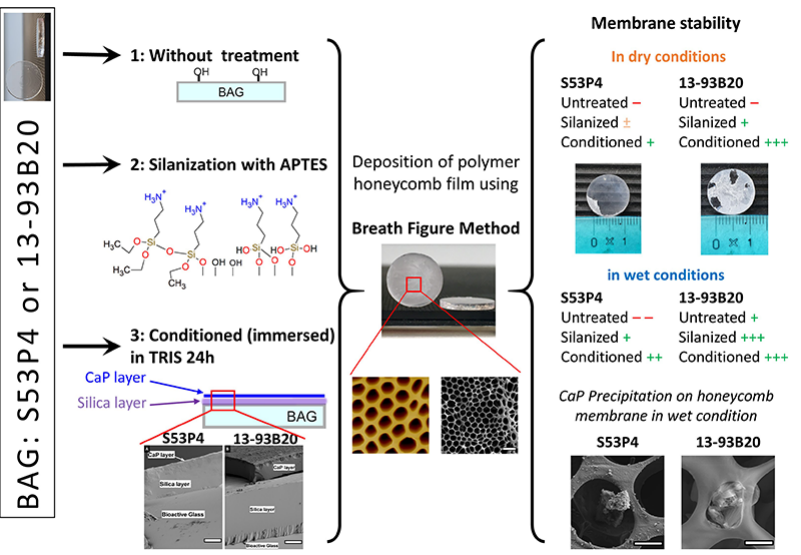

The development of innovative materials for bone tissue engineering to promote bone regeneration while avoiding fibrous tissue infiltration is of paramount importance. Here, we combined the known osteopromotive properties of bioactive glasses (BaGs) with the biodegradability, biocompatibility, and ease to shape/handle of poly-l-*co*-d,l-lactic acid (PLDLA) into a single biphasic material. The aim of this work was to unravel the role of the surface chemistry and topography of BaG surfaces on the stability of a PLDLA honeycomb membrane, in dry and wet conditions. The PLDLA honeycomb membrane was deposited using the breath figure method (BFM) on the surface of untreated BaG discs (S53P4 and 13-93B20), silanized with 3-aminopropyltriethoxysilane (APTES) or conditioned (immersed for 24 h in TRIS buffer solution). The PLDLA membranes deposited onto the BaG discs, regardless of their composition or surface treatments, exhibited a honeycomb-like structure with pore diameter ranging from 1 to 5 μm. The presence of positively charged amine groups (APTES grafting) or the precipitation of a CaP layer (conditioned) significantly improved the membrane resistance to shear as well as its stability upon immersion in the TRIS buffer solution. The obtained results demonstrated that the careful control of the substrate surface chemistry enabled the deposition of a stable honeycomb membrane at their surface. This constitutes a first step toward the development of new biphasic materials enabling osteostimulation (BaG) while preventing migration of fibrous tissue inside the bone defect (honeycomb polymer membrane).

## 1 Introduction

It is commonly accepted that bone tissue regeneration requires innovative materials, with various properties, i.e., biocompatibility, osteoconductivity/osteoinductivity, while promoting angiogenesis.^1−3^ In addition, newly developed biomaterials should have a structural organization mimicking the natural bone. One challenge that is often encountered when using bone grafts (natural or synthetic) is the invasion of implants by soft/fibrous tissue before proper bone regeneration occurs. This is due to the faster proliferation rate of cells involved in the wound healing process (e.g., fibroblasts) compared to that of the bone cells.^4^ Therefore, invasion of the bone defect by soft tissue will ultimately lead to incomplete bone regeneration.^5,6^ To prevent this negative outcome, membranes have been used to cover the bone defect and thus prevent fibrous tissue ingrowth.^5,7^ Many types of membranes have been developed, either made from synthetic polymers (either degradable, i.e., aliphatic acids such as poly-l-lactic acid (PLLA), poly-l-lactide-*co*-glycolide (PLGA) or not degradable such as polytetrafluorethylene (PTFE)) or natural polymers (collagen or chitosan, for example).^5,8^ As of today, the majority of commercially available membranes are based on synthetic degradable polymers or collagen.^9^ These membranes exhibit high biocompatibility, favor cell adhesion, and do not necessitate to be retrieved during a second surgery. However, they have an unpredictable degradation rate, leading to a mismatch between the membrane degradation and the new bone formation rate.^9^ There is still important work to be done to achieve the production of the ideal protective membrane, but there is a consensus on their required properties. The ideal barrier membrane should (1) be biocompatible, (2) be cell-occlusive, (3) allow space-making (“define the volume of bone that can be regenerated”^10^), (4) allow tissue integration, (5) be easy to handle, and (6) have an appropriate pore size and pore interconnectivity to facilitate bone regeneration but preventing excessive fibrous tissue penetration.^5,10−12^ While initially the membrane was only used to direct the bone regeneration (without the use of bone grafts), the review by Dimitriou et al.^5^ reports the use of barrier membranes associated with a bone graft (natural or synthetic) since the early 2000s. Since then, researchers have focused on understanding the impact of using a membrane in addition to the bone graft on bone regeneration.^13−16^ In such cases, the membrane and the graft are two materials that are not in direct contact. While the use of a membrane alone protects the defect from fibrous tissue ingrowth, the addition of a bone graft underneath the membrane was associated with a faster bone regeneration.^16−18^

In the present study, a proof of concept for a new biphasic material where a biodegradable polymer-based barrier membrane was directly deposited on a synthetic osteostimulative substrate is proposed for the first time, to the best of the authors’ knowledge. One phase, made of a honeycomb-structured poly-l-*co*-d,l-lactic acid (PLDLA) barrier membrane, providing protection from fibrous tissue ingrowth while still allowing exchange of ions and nutrients and a second phase, made of dense bioactive glasses (BaG), promoting bone regeneration. Indeed, such approach could allow the design of patient-specific graft providing a 2 in 1 solution, easy to use, in complex surgery for large bone defect (e.g., mandibulectomy, wide palatal defect, etc.). PLDLA was chosen as the material forming the barrier membrane. As mentioned previously, membrane porosity must be carefully controlled as it is one of the key factors to achieve good tissue integration while avoiding fibrous tissue ingrowth. One successful method to control the membrane porosity is the breath figure method (BFM).^19^ This method allowed us to create highly organized honeycomb-like porous surfaces through a simple process. In short, (1) the desired polymer is mixed with a volatile water-immiscible solvent, (2) the solution is cast on a substrate under a high relative humidity (RH) airflow which allows water condensation at the polymer solution surface, while the solvent evaporates (3) when water and solvent have completely evaporated, a membrane with a highly ordered porous surface is formed.^19,20^ Its low cost and its ease of implementation make the BFM a widely used method to produce porous polymer membranes.^19,21^ Furthermore, it has been shown that membranes prepared using BFM and having appropriate pore sizes can adequately support cell adhesion and proliferation.^21−23^ In addition, in this study, BaG was chosen as the substrate onto which the membrane was deposited. BaGs have been extensively studied for their ability to promote osteoconduction or even osteoinduction.^24,25^ The composition of BaGs can be tailored, to ensure the release of the most therapeutically relevant ions for the intended application.^26^ Over the years, BaGs have been found to be osteostimulative, to favor angiogenesis,^27^ and to have antimicrobial properties.^28,29^ Due to their high interest in bone regeneration, the surface chemistry of BaGs, as well as their ability to be functionalized in view of increasing the adsorption rate of biomolecules or to increase the connectivity between the glass and the polymeric phase, have been widely studied.^30−33^

In this manuscript, we reported the deposition of a PLDLA membrane, processed by BFM, onto a bioactive glass. PLDLA was chosen for its ease of processing into a honeycomb membrane with controlled surface porosity,^19,34^ while BaG was used for its bioactivity. Two substrates have been studied, i.e., S53P4 and 13-93B20. The S53P4, also known as BoneAlive S53P4, is a well-known and widely used silicate BaG which has the US Food and Drug Administration approval,^35,36^ while the glass 13-93B20 is an experimental glass composition already reported as part of composites in ref (37). The impact of substrate surface physicochemical properties (surface charge, ion release, etc.) on the interfacial stability of the membrane was assessed. The aim of this work is to design a promising biphasic material that can retain its bioactivity (through controlled ion release) while maintaining the membrane integrity. The controlled pore size of the membrane and its stability over time will expectedly allow ion transfer while preventing fibroblasts from migrating within the graft.

## 2 Materials and Methods

### 2.1 BaG Material Synthesis and Surface Treatments

S53P4 and 13-93B20 BaG were prepared from analytical grade K_2_CO_3_ (Alfa Aesar, Thermo Fischer, Kandel, Germany), Na_2_CO_3_, NH_4_H_2_PO_4_, (CaHPO_4_)(2(H_2_O)), CaCO_3_, MgO, H_3_BO_3_ (Sigma-Aldrich, Saint-Louis, MS), and Belgian quartz sand. The nominal oxide compositions of the experimental BaGs are presented in Table 1 in mol %.

**Table 1:** Composition of the BaGs in mol %

	mol %						
glass	Na_2_O	CaO	P_2_O_5_	SiO_2_	K_2_O	MgO	B_2_O_3_
S53P4	22.66	21.77	1.72	53.85			
13-93B20	6.0	22.1	1.7	43.7	7.9	7.7	10.9

The reagents were melted in a platinum crucible at 1450 °C in an electrical furnace. The molten glass was then cast into a preheated graphite mold to obtain a rod with a diameter of 14 mm. The glass rods were then annealed overnight at 500 °C and let to cool down to room temperature. The rods were then cut into 2 mm thick discs and polished with SiC paper (grit #320, #500, #800, #1200, #2400, and #4000, from Struers, Copenhagen, Denmark). All samples were dried and kept in a desiccator until further use.

Membranes were directly deposited onto untreated or surface-modified BaG discs. Discs with both BaGs composition were surface treated by either silanization or conditioning. The surface treatment protocols are as follows.

#### 2.1.1 Silanization with 3-Aminopropyltriethoxysilane (APTES)

Polished BaG discs were silanized with 3-aminopropyltriethoxysilane (APTES) (Thermo Fischer Scientific, Germany), according to the protocol used by Massera et al.^38^ Briefly, the BaG discs were first washed for 5 min in acetone and distilled water (three times), in a sonicating bath. After washing, the BaG discs were immersed in ethanol (150 mL) with APTES (70 μL) for 6 h and, successively, dried at 100 °C for 1 h. To remove the loosely bound APTES, the BaG discs were then washed again in ethanol for 5 min in the sonicating bath and further dried for 30 min at 100 °C.

#### 2.1.2 Conditioning

Polished BaG discs were immersed in TRIS buffer solution and incubated at 37 °C for 24 h. TRIS solution was prepared from Trisma base and Trisma HCl (Sigma-Aldrich, Saint-Louis, MS) at pH 7.38 ± 0.02 at 37 ± 0.2 °C. After incubation, the solution was removed, and the BaG discs were allowed to dry in a fume hood overnight before membrane deposition.

### 2.2 Honeycomb Membrane Deposition

Honeycomb membranes were fabricated from a 10 mg·mL^–1^ solution of 96/04 l-lactide/d-lactide copolymer (PLDLA) containing 0.1 mg·mL^–1^ of the surfactant dioleoyl phosphatidylethanolamine (DOPE) in chloroform. PLDLA purified, medical grade, PURASORB PLD 9620 was purchased from Corbion Purac, The Netherlands and DOPE from Sigma-Aldrich, Japan.

**Figure 1:**
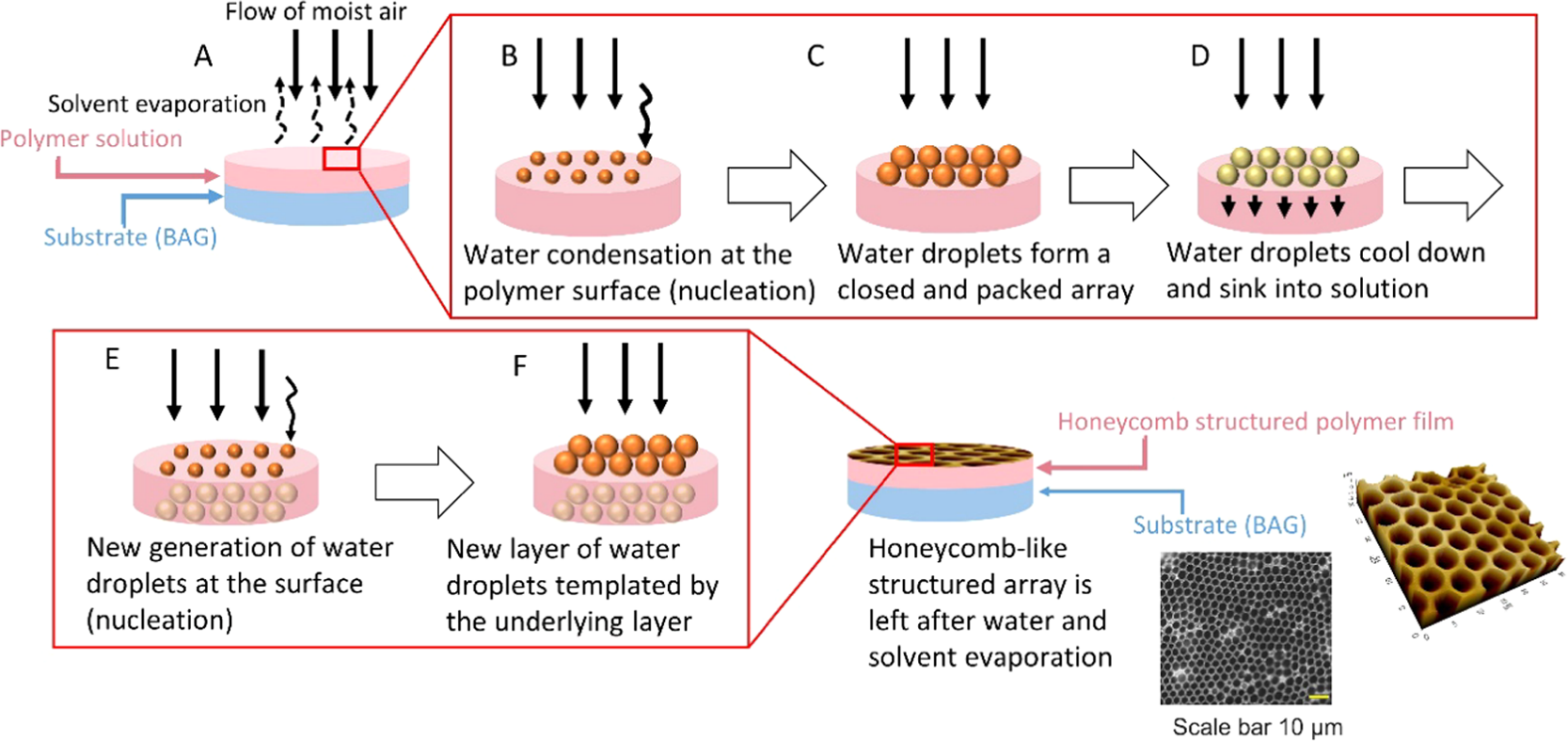
Schematic of the membrane deposition process, using the BFM. (A) Deposition of the polymer solution on the substrate (BaG) and placing the construct under a flow of moist air, (B) water droplets start to condense at the surface of the polymer solution, (C) water droplets grow and form a closed and packed array, (D) droplets cool down and sink into the solution, (E) new generation of water droplets is formed at the surface, (F) process continues until the end of the reaction under the flow of moist air, and each new generation of water droplets is templated by the underlying layer.

The honeycomb membranes were produced by the BFM as described in Figure 1 and as previously reported in ref (19). Briefly, the polymer solution was deposited drop by drop onto BaG discs (untreated, silanized, and conditioned) and then the solvent was allowed to evaporate in a humidity chamber at 80 ± 5% RH, under airflow. The samples were air-dried at room temperature and then washed twice with 70% ethanol to remove the surfactant. Samples were air-dried again and stored in a desiccator until further use.

### 2.3 Material Characterization

#### 2.3.1 ζ-Potential

An electrokinetic analyzer for solid surfaces (SurPASS 3, Anton Paar, Austria) was employed to measure the ζ-potential of untreated and treated BaG discs by means of the streaming potential technique.^39^ An adjustable gap cell was used for the measurements, and a 1 mM KCl solution was used as the electrolyte. Measurements were carried out at pH = 7.

#### 2.3.2 Shear Stress Test

Two aluminum plates were clamped to a TA1 texture analyzer (Lloyd materials testing, AMETEK, Pennsylvania) equipped with a 20 or 100 N load cell, depending on the force to be applied. The specimen to be tested was fixed in-between the plates, by solvent-free double-sided tape (tesa ECO FIXATION). Freshly prepared samples were used for the measurement. Shear force on the membrane was created by pulling the upper plate at 1 mm·min^–1^ while the bottom aluminum plate remained fixed. The design of the setup can be found in ref (40). The test was performed on five to seven samples.

#### 2.3.3 BaG Disc Surface Topography and Composition

Scanning electron microscopy–energy-dispersive X-ray spectroscopy analysis (SEM/EDX) was conducted using a Gemini SEM 300 (Carl Zeiss, Germany) equipped with an EDS Bruker Quantax (Bruker) for EDX spectroscopy. Samples were metalized with nickel (for EDX) 4 times 30 s at 30 mA (for EDX analysis) or with a 4 nm thick platinum layer using a Leica ACE600 (Leica, Wetzlar, Germany) (for SEM imaging).

#### 2.3.4 Structural Property

The infrared (IR) absorption spectra of untreated or treated BaG discs were recorded using a Bruker Alpha FTIR in attenuated total reflectance (ATR), to see the effect of treatments on their surface chemical properties. All IR spectra were recorded within the range 400–4000 cm^–1^ with a resolution of 2 cm^–1^ and 64 accumulation scans. All spectra were corrected for Fresnel losses and normalized to the band with maximum intensity.

#### 2.3.5 Stability Tests

The membrane stability was studied in dry and wet conditions.

##### 2.3.5.1 In Dry Conditions

Samples (*n* = 3) were dried and kept at room temperature in a desiccator (20–40% RH) inside multiwell plates for up to 4 weeks. Topographical features of honeycomb films were analyzed using an atomic force microscope (AFM) XE-100 Park System Corp. An image size of 30 μm × 30 μm was scanned in noncontact mode, under air and at room temperature. Acquired images were analyzed using image analysis software (XEI, Park System). The pore size was estimated from the AFM images using the software Fiji.

##### 2.3.5.2 In Wet Conditions

Samples (*n* = 12) were immersed in 5 mL of TRIS buffer solution before being incubated at 37 °C in static conditions (without agitation). The buffer solution was refreshed at 3, 6, 24, 48 h, 5, 7, 9, 14, and 21 days to prevent saturation of the immersion solution with ions released from the BaG substrate. The assembly (membrane/BaG disc) integrity was assessed by counting the number of membranes that detached (partially or totally from the substrate) during the immersion period. At 28 days (4 weeks), samples were collected and left to dry in a fume hood overnight before further analysis.

All samples were imaged by AFM and SEM/EDX, as described above.

At each time point (3, 6, 24, 48 h, 5, 7, 9, 14, 21, and 28 days), 1 mL of the immersion solution was collected to quantify the change in ion concentration over the incubation period. Inductively coupled plasma-optical emission spectroscopy (ICP-OES) analysis was conducted with an Agilent 5110 instrument (Agilent technologies) equipped with a SPS 4 autosampler, to quantify the presence of phosphorus (P), sodium (Na), calcium (Ca), silicate (Si) (for both BaGs) and boron (B), potassium (K) and magnesium (Mg) (only for 13-93B20) in the medium collected during the immersion in TRIS buffer solution. Wavelength values for the analysis were as follows: P, 213.618 nm; Na, 589.592 nm; Ca, 317.933 nm; Si, 250.690 nm; B, 249.678 nm; K, 766.491 nm, and Mg, 279.800 nm.

## 3 Results and Discussion

Materials were first studied in dry conditions to assess the impact of aging on the adhesion of membranes to the substrates. Samples were subsequently immersed to observe and understand the degradation process of the materials in aqueous conditions.

### 3.1 BaG Disc Treatment, Deposition, and Characterization of the Stability of Membranes in Dry Conditions

#### 3.1.1 Surface Treatments

First, the impact of the treatment on the surface charge of BaG discs was analyzed. ζ-Potential measurements are reported in Table 2.

**Table 2:** ζ-Potential of Untreated, Silanized, and Conditioned BaG Disc Surfaces at pH 7 (Streaming Potential)

	S53P4			13-93B20		
	untreated	silanized	conditioned	untreated	silanized	conditioned
ζ-potential (mV)	–47.8 ± 0.5	–30.6 ± 2.0	–16.9 ± 0.4	–53.2 ± 1.9	–12.2 ± 0.4	–15.5 ± 0.4

As expected, with ζ-potential around −50 mV, the surface charge of the untreated samples is in agreement with the values for silicate and borosilicate glasses.^41,42^ Regardless of the BaG composition, both treatments (silanized and conditioned) led to a decrease in the surface charge. In the case of silanization with APTES, the decrease in surface charge can be explained by the introduction of positively charged amine groups to the BaG disc surface at pH = 7.^41^ Upon conditioning for 24 h in TRIS buffer solution, the BaG discs started to dissolve which resulted in the formation of Si–OH and Si–O^–^ groups on their surfaces. Eventually, if the dissolution/reaction in an aqueous solution is rapid, a calcium phosphate reactive layer may start to precipitate.^35,43^ Using a silicate glass model, Lu et al. reported that during immersion the measured ζ-potential presents a shift toward positive values, corresponding to the formation of an amorphous Ca–P layer, which can be detected as early as 1 day after immersion.^44^ At longer immersion times, amorphous Ca–P layers crystallize. The crystalline hydroxyapatite layer has been reported to have a ζ-potential value close to −15 mV.^43,45^ Based on these results, the surface charge decrease observed in our study may be explained by (1) the density of positively charged amine groups at the surface of silanized samples and (2) the nature (composition, specific surface area) of the Ca–P layer that has possibly deposited during the preincubation of the BaG discs for 24 h.

When comparing BaG compositions, it was clear that the surface charge of untreated and conditioned glass discs, respectively, was similar. However, silanization with APTES was found more efficient in reducing the electronegativity on the glass 13-93B20 than on the glass S53P4. Such variation in the surface charge between BaGs might be correlated with their dissolution rates. Indeed, borosilicate BaGs are known to possess a borate phase with higher reactivity than silicate BaGs.^46,47^ Such a fast, early dissolution may lead to an increase in the density of Si–OH groups that are formed during the washing step, in turn leading to a higher density of sites onto which the APTES can be attached. The higher the concentration of amine groups, the less negative the surface will be. Indeed, Ferraris et al. have reported that upon silanization, the increase of the ζ-potential is dependent on the density of amine groups.^41^ Therefore, the smaller change in surface charge seen for the S53P4 glass when compared to the 13-93B20 glass can be assigned to a greater density of positively charged amine groups at the surface of the latter composition. However, one should keep in mind that the dynamic dissolution of the BaG may also lead to the release of amine groups.

**Figure 2:**
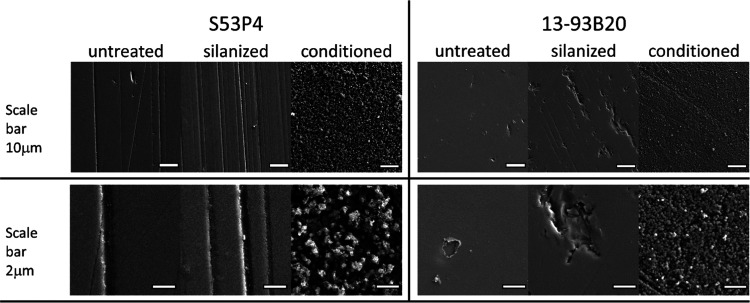
SEM images of the surface of untreated, silanized, and conditioned BaG discs, before membrane deposition.

**Figure 3:**
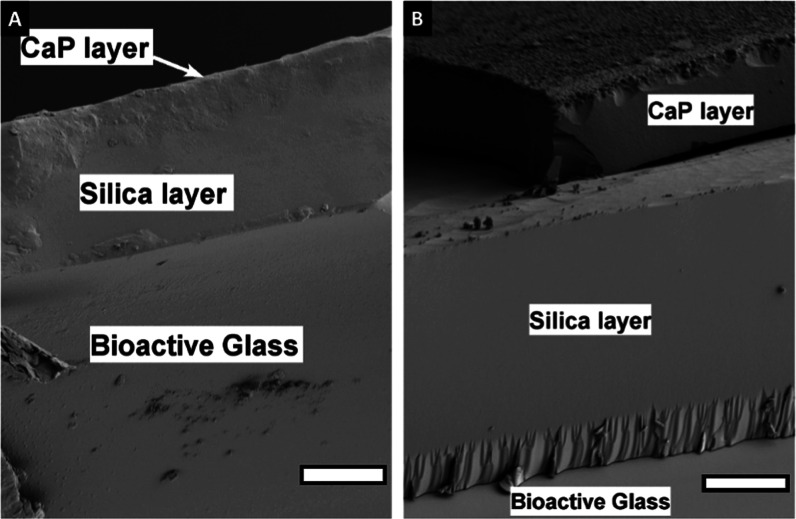
SEM images of cross section of S53P4 (A) and 13-93B20 (B) conditioned analyzed by EDX, scale bar: 20 μm.

To obtain more information on the surface texture of different BaG discs and the impact of treatments on the surface composition, BaG discs were imaged by SEM/EDX (Figures 2 and 3).

**Figure 4:**
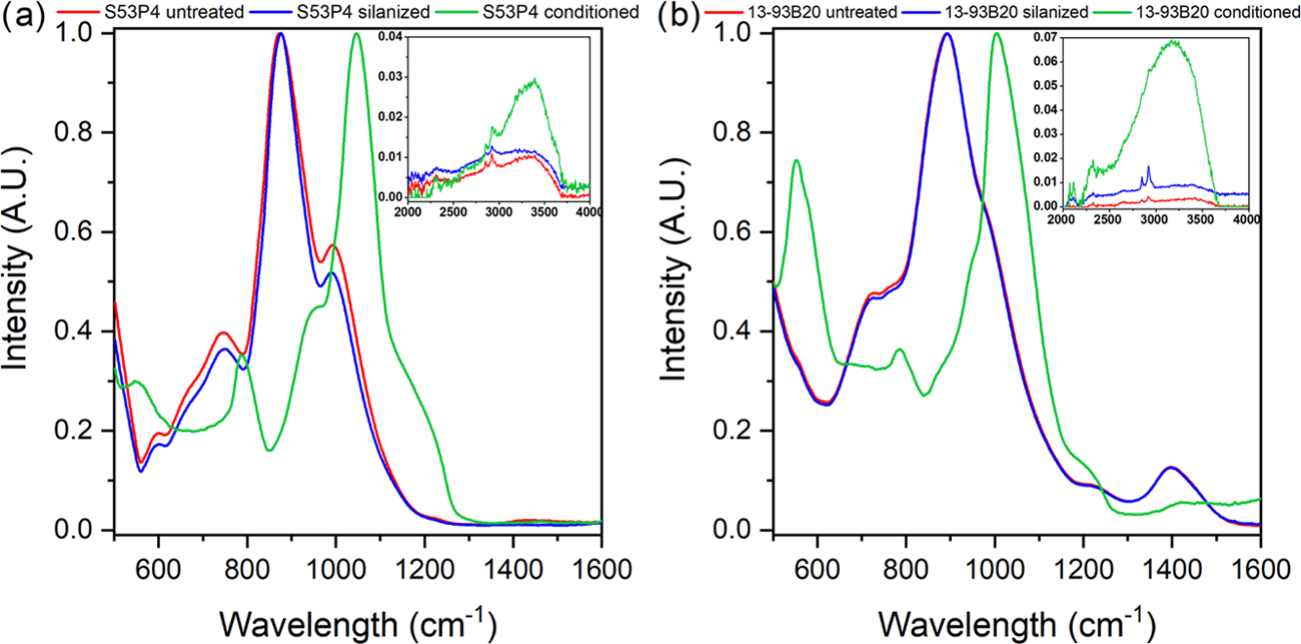
FTIR-ATR spectra of S53P4 (a) or 13-93B20 (b), untreated (red), silanized (blue), and conditioned (green) prior to membrane deposition. The inset in each spectrum shows the 2000-4000 cm^–1^ region.

At the microscopic level, silanization of S53P4 does not seem to have a significant impact on surface texture, whereas in the case of 13-93B20, the signs of surface degradation can be seen. In addition, a high density of nodules with sub-micrometer size can be observed on conditioned BaG discs. At higher magnification, one can see that nodules are smaller and denser at the surface of 13-93B20 than at the surface of S53P4. The cross section of samples was analyzed by EDX (Figure 3) and the top surface by Fourier transform infrared (FTIR) spectroscopy (Figure 4).

SEM/EDX analysis of conditioned samples indicated the presence of three phases: (1) the bioactive glass, (2) a silica-rich layer, and (3) a reactive layer composed mainly of Ca and P. The Ca/P ratio was found to vary between 1.4 and 1.7, regardless of the BaG composition. The large variation in the ratio can be assigned to the (1) high penetration depth of the electron beam (signal from the underneath BaG is collected) and (2) the Ca deficiency of the early apatite layer formed at the surface of BaG.^42^ The formation of such layers was expected upon immersion of silicate and borosilicate BaGs into aqueous solutions.^46−48^ It is interesting to point out that the reactive layer at the surface of S53P4 glass had a lower density of nodules than the surface of 13-93B20 (Figure 3). Such a thin layer at the surface of S53P4, formed upon immersion in TRIS buffer solution, was also reported before by Varila et al.^49^

The FTIR-ATR spectra of the top layer are presented in Figure 4.

The FTIR-ATR analysis was made to identify the chemical structure at the surface of the glasses.

FTIR-ATR spectra of untreated S53P4 (Figure 4a) and 13-93B20 (Figure 4b) displayed bands ∼748, ∼930, and ∼1030 cm^–1^. These bands can be attributed to Si–O bending, Si–O^–^ (nonbridging oxygen) in the [SiO_4_] units, and to Si–O–Si asymmetric stretching in [SiO_4_] units, respectively.^50,51^ Aside from those bands, the glass 13-93B20 also exhibited bands at 1400 cm^–1^ related to BO_3_ vibrations.^51,52^ Silanization did not seem to significantly impact the surface chemistry, regardless of the glass composition. While vibration related to amine groups (NH_2_ between 1400 and 1600 cm^–1^) could be expected, they were not visible in the FTIR-ATR spectra of silanized BaG discs. The reason may lie in the low density of amine groups at the surface of the BaG discs.^38,41^ However, as an amine group signal is visible in the same region as BO_3_ units in 13-93B20, it is possible that those bands were covered by boron bands in this glass.

Major changes in the surface structure occurred for conditioned BaG discs, as expected from SEM/EDX. The FTIR-ATR spectra of conditioned samples exhibited complete disappearance of vibration bands related to silicate and borate networks and new absorption bands at ∼560, ∼605, ∼800, and ∼1060 cm^–1^ as well as a shoulder at ∼959 cm^–1^ appeared. The shoulder at ∼959 cm^–1^ can be attributed to C–O vibration mode in CO_3_^2–^ and to P–O–P bonding.^50^ The bands at ∼800 and ∼1060 cm^–1^ can be assigned to the C–O bending and P–O stretching vibration, respectively.^53^ Bands at ∼560 and ∼605 cm^–1^, in the conditioned BaG disc spectra, attributed to the P–O resonance of PO_4_^3–^, were characteristic of an apatite structure.^48^ Furthermore, conditioned samples presented a band of higher intensity in the region 3000–3600 cm^–1^ corresponding to OH vibration indicating a hydrated layer at BaG disc surfaces (Figure 4a,b insets).^38^

These spectra confirmed the presence of a hydroxyapatite layer at the surface of conditioned BaG discs and revealed that there were no significant differences in the surface chemistry of silanized and untreated BaG discs.

#### 3.1.2 Deposition of PLDLA Honeycomb Membrane

**Figure 5:**
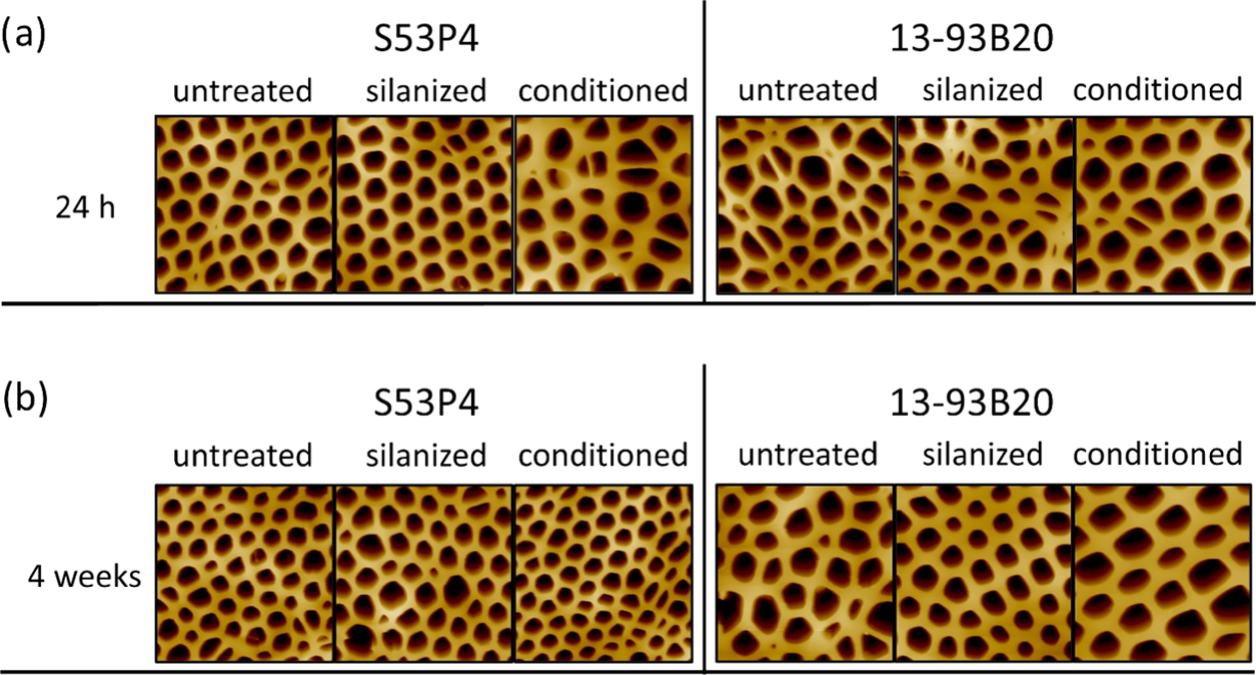
AFM images of the membranes deposited on the different substrates 24 h (a) or 4 weeks (b) after aging in a desiccator at 40% RH (each image is 30 μm × 30 μm).

Figure 5 presents the AFM images of the membranes deposited on different BaG discs (untreated and treated). The images, taken 24 h postdeposition (Figure 5a), allowed us to assess the relationship between the physicochemical features of different BaG disc surfaces and the features of the membranes prepared by the BFM.

After 24 h aging (Figure 5a), regardless of the substrate, a honeycomb-like pattern was always visible, in spite of some variation in the homogeneity of pores. The pore area was calculated and found to be 5–20 μm^2^ (data not shown), and the thickness of the membrane was found to vary from 10 to 20 μm. Assuming that pores had a shape close to a circle, this corresponded to a diameter of 1–5 μm, which was similar to the values reported in the literature for PLDLA honeycomb membranes.^19^ It is well known that when using the BFM, small variations in the humidity, in the viscosity of the polymer solution or in room temperature, can greatly influence the final shape of the honeycomb.^19,21,54^

#### 3.1.3 PLDLA Membrane Resistance and Stability in Dry Conditions

The attachment of the membrane to its substrate was then evaluated by applying a shear stress on the materials and by measuring the force needed to detach the membranes. The results are shown in Table 3.

**Table 3:**
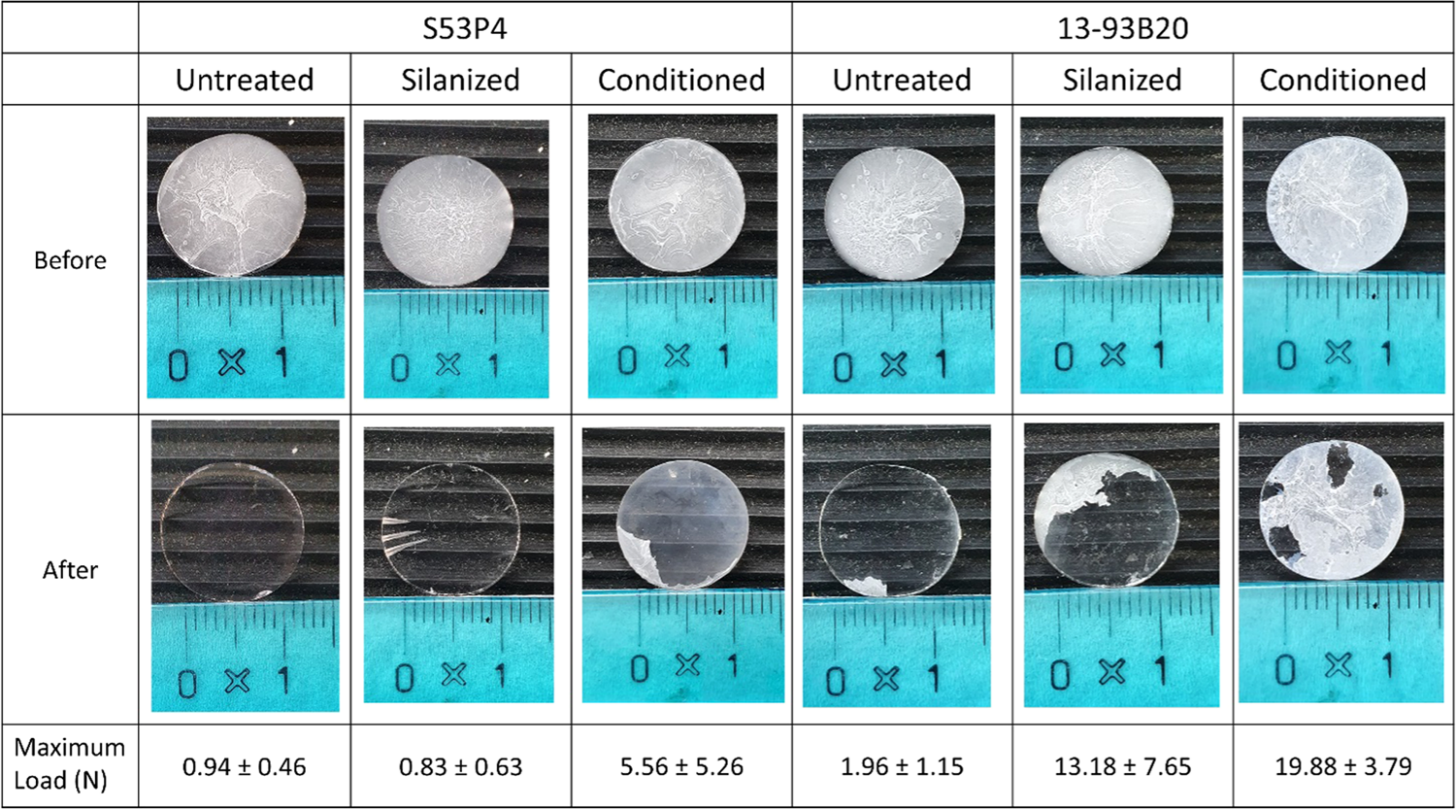
Photographs of the PLDLA Membrane Deposited on BaG Discs before (Upper Row) and after (Lower Row) the Shear Stress Test^a^

a Upon shear, the loss of the membrane is revealed by the appearance of the transparent glass substrate.

The results showed that the membranes deposited on untreated and silanized S53P4 substrates exhibited full detachment from the glass surface. In the images, almost no residues of the membrane were visible on the glass surface with a maximum load inferior to 1 N. On the other hand, the membranes deposited on the conditioned S53P4 detached only partially, and the force needed to detach them was more than 5 times higher than that needed to detach the membrane from silanized and untreated S53P4.

In the case of 13-93B20, untreated and silanized BaGs behaved similarly, i.e., part of the membrane detached from the substrate, but some residues were observable after the test. In contrast with S53P4, silanization of 13-93B20 greatly increased the resistance to shear (more than 10 times). The attachment strength of the membranes deposited on the conditioned 13-93B20 outperformed all the other substrates and treatments. In spite of the membranes becoming mildly damaged following a maximum load of 19.88 N, a large portion of the membranes remained tightly attached to the substrate after the test, with the shear force needed to achieve detachment being greater than for all other samples. It is noteworthy that, in all the cases, the standard deviation indicated a high degree of inhomogeneity between samples. Inhomogeneities on untreated samples can be attributed to small differences in the surface finish of the postpolishing of the samples. In the case of silanized samples, differences may arise from the APTES physisorption. While the exposure of amine groups was the most likely event, one cannot overlook the possibility of the APTES being bound to the BaG disc surface by the amine group, thus revealing ethoxy groups.^55^ Upon conditioning, the texture, topography, and density of reactive layer across the surface of the disc cannot be precisely controlled, especially in the case of S53P4 where the precipitation was less prominent than for 13-93B20. Finally, as mentioned earlier, a small variation in membrane deposition parameters (i.e., temperature, humidity, etc.) can lead to small changes in membrane properties.^19^

The stability of membranes in dry conditions as a function of time and without external stress was also studied. Membranes deposited onto various BaG disc surfaces were imaged using the AFM, 4 weeks postdeposition, as shown in Figure 5b. When compared to Figure 5a, the honeycomb structure kept its integrity for at least 4 weeks in a dry environment (desiccator). Most of the pores were found in the range of 1–5 μm in diameter. As stated above, a large variability in the pore dimension was measured, which does not seem to be correlated with membrane aging nor with the treatment applied to the substrate, but rather with the processing methods and variables (humidity, polymer solution viscosity, temperature).

### 3.2 Stability of the Membrane/BaG Disc Assembly in Aqueous Conditions

#### 3.2.1 Assembly Integrity in Aqueous Solution

**Figure 6:**
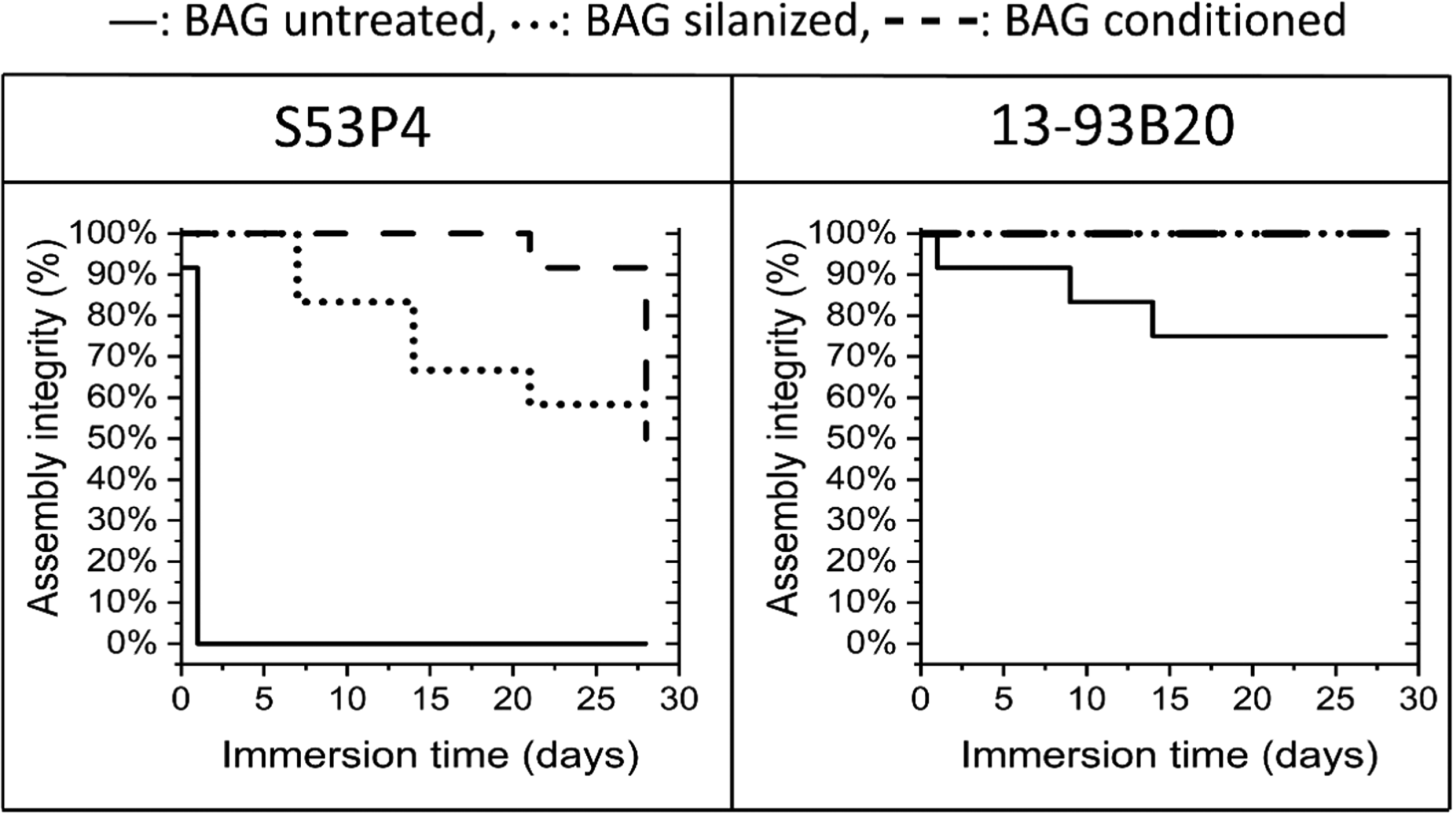
Assembly integrity (in %) was estimated by counting the number of membranes that did not detach (partially or totally) from their substrate, as a function of immersion time, *n* = 12.

The stability of the membrane/BaG disc assembly was then studied by immersing the material in TRIS buffer solution at 37 °C, for up to 4 weeks (Figure 6).

All membranes deposited on untreated S53P4 detached after 3 h of immersion. Membranes started to detach after 7 and 21 days on S53P4 silanized and conditioned, respectively. Compared to untreated S53P4, membranes deposited on untreated 13-93B20 were noticeably more stable. Indeed, 70% were still attached to their substrate after 28 days of immersion. While borosilicate glass is typically considered more hydrolytically unstable than silicate glass, this is solely due to the borate phase which degrades at a faster rate than the silicate phase.^51^ As per the FTIR-ATR spectra in Figure 4, one can see that the silicate network in the S53P4 glass has a greater number of nonbridging oxygen (ratio between the bands at ∼930 and ∼1030 cm^–1^) than the silicate network in the 13-93B20 glass.^56^ Therefore, the initial dissolution of the SiO_2_ network occurs faster for the S53P4 glass, leading to a decreased interface stability between the glass and the membrane.

Silanization improved drastically the assembly integrity, regardless of the BaG composition. It is interesting to note that membranes deposited on silanized S53P4 seemed to detach gradually over time. Sixty percent of the membranes remained attached to the substrate after 4 weeks of immersion, while 100% of the membranes were still attached to their substrate on silanized 13-93B20. As per the ζ-potential, it is believed that the surface of 13-93B20 was grafted with a higher density of amine groups leading to a higher stability of the membrane at the glass surface. Zhou et al. reported interactions between PLDLA and hydroxyapatite, thereby hydrogen bonds form between C=O and P-OH functions.^57^ Similarly, in this study, it is feasible that amines and the C=O group interact through hydrogen bonding.

Finally, on the conditioned S53P4, membranes remained stably attached to the substrate for 20 days, with 50% of the membranes abruptly detaching at 27 days. Membrane attachment was found to be significantly improved when the conditioned 13-93B20 BaG was used as the substrate, with 100% of the membranes remaining attached at the end of the immersion period. As shown by the SEM/EDX (Figures 2 and 3) and FTIR-ATR analysis (Figure 4), the surface chemistry has changed during the immersion for 24 h in TRIS buffer solution, thereby a Ca–P reactive layer has formed at the surface of the glass. This is believed to be the reason for the stability of the assembly upon immersion.

ζ-Potential, mechanical testing, and immersion into TRIS buffer solution indicated that:

(1) The stability of the membrane was highly dependent on the surface reactivity, i.e., in solution, the more reactive surface will lead to a faster failure of the membrane.
(2) Silanization improved the stability of the membrane/BaG disc assembly in an aqueous solution. The improvement was a function of the amine group density (i.e., surface charge). However, only at higher silanization density, an increased shear stress is necessary to detach the membrane from the substrate (i.e., for silanized 13-93B20, Table 3).
(3) Membranes deposited on conditioned samples demonstrated improved resistance to shear, as well as higher stability in aqueous solutions. Such improvement in the membrane/BaG disc assembly stability was linked to the precipitation of a stable Ca–P reactive layer. The thicker the layer, the more stable the membrane, probably due to an increased specific surface area and/or interactions between the hydroxy groups of the reactive layer and carbonyl groups of the polymer.^57^ The impact of the specific surface area on the membrane adhesion will be studied in the future.

Overall, a controlled surface treatment of bioactive substrates led to an improvement in the assembly integrity. This is of paramount importance in view of culturing cells without the risk of the membrane detaching over time. Furthermore, when thinking of the application (i.e., a biphasic bone substitute), proper adhesion of the membrane to its substrate is crucial, up until the time the defects have been repaired.

#### 3.2.2 BaG Ion Release, from the Assembly, in Aqueous Solution

**Figure 7:**
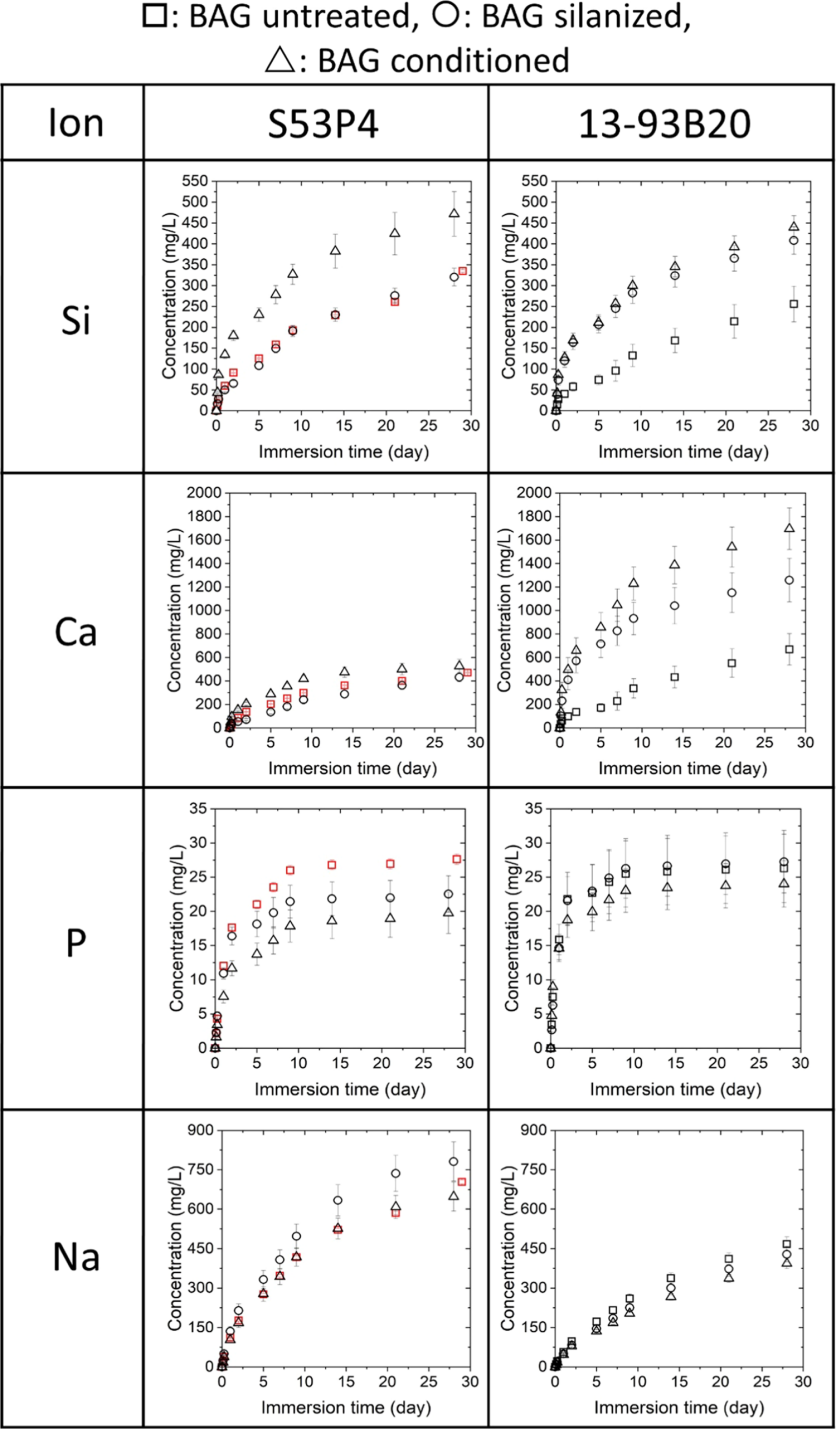
Silicon (Si), calcium (Ca), phosphorous (P), and sodium (Na) release profile upon immersion of the membrane/BaG disc assembly in TRIS buffer solution for up to 28 days. Red squares display the results of untreated S53P4 without a membrane.

**Figure 8:**
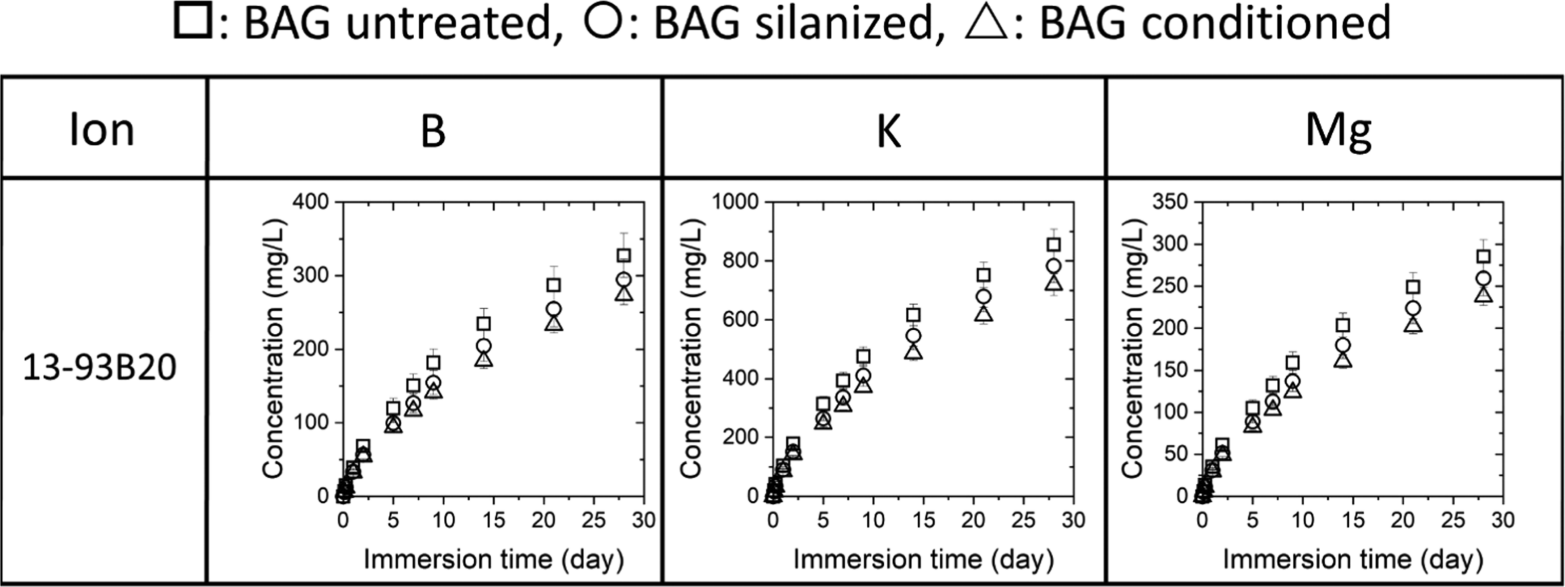
Ion release profile of boron (B), potassium (K), and magnesium (Mg) for the three 13-93B20-containing membrane/BaG disc assembly as a function of immersion time in TRIS buffer solution.

It is well known that BaGs react and release ions upon immersion, which can have beneficial effects on cell fate.^25,52^ The release profile of Si, Ca, P, and Na ions by both BaGs is presented in Figure 7, while the release profile of B, K, and Mg ions, specific to the composition of the 13-93B20 glass, is shown in Figure 8.

The ion release profiles for untreated samples are also reported in the figures. However, membranes deposited on untreated S53P4 were not studied further, due to their poor stability in aqueous conditions (Figure 7, all membranes detaching after 3 h). Therefore, the ion release from this material does not reflect the release rate of ions through the membrane but rather from the substrate alone. The data are included to allow for comparison in dissolution kinetics between the various treatments on S53P4.

As suspected, the release of Si from untreated S53P4 was slightly faster than the release rate observed for untreated 13-93B20, which confirmed that the decreased membrane stability in the aqueous solution was probably due to the rapid release of ions from the glass surface. A faster Si release from S53P4, when compared to 13-93B20, was expected. Indeed, BaG 13-93B20 was developed by substituting 20% of SiO_2_ with B_2_O_3_ in the silicate glass 13-93.^37^ The silica network, in the glass 13-93 (without boron), is more polymerized than in S53P4 and therefore 13-93 is more stable to hydrolysis.^58^ In addition, the partial substitution of B_2_O_3_ for SiO_2_, in 13-93B20, further leads to an increased polymerization of the SiO_2_ network making 13-93B20 silica network less sensitive to hydrolysis compared to S53P4.^37,59^ Upon silanization, one can see that the Si release for S53P4 did not significantly change, whereas it increased for 13-93B20. This can be assigned to the pretreatment of the materials during silanization and/or release of Si from the grafted APTES. Finally, the conditioned S53P4 BaG released more Si than the silanized counterpart, whereas the Si release profile from the conditioned 13-93B20 was similar to the Si release from the silanized 13-93B20 material. The greater Si release from the conditioned S53P4 compared to 13-93B20 can be explained by the change in surface chemistry. Indeed, as the reactive layer was thinner on S53P4 BaG, more silica gel was in contact with the solution, in turn leading to higher Si release to the surrounding medium. It is important to note that after 3 days of immersion, the silicon release seemed to slow down. This phenomenon is in agreement with previous studies discussing the Si release from BaGs.^41^

The phosphorous release profile was similar for all BaGs. Phosphate concentration seemed to saturate, as soon as 1 week for all samples. The shape of the curve indicated that phosphate release followed a typical diffusion-controlled process. However, as the results are cumulative, this could also indicate the saturation of the solution with phosphate ions, leading to precipitation of a reactive layer.^60^ The phosphorous release profile appeared to be independent of the surface treatment in 13-93B20. However, untreated S53P4 released more phosphorous than the surface-treated ones. This can be attributed to the absence of the membrane in this particular condition.

Sodium release from S53P4 and 13-93B20 glass samples was consistent with the dissolution mechanism described by Hench^24,61^ for BaGs. Indeed, the conditioned samples seemed to release Na at a lower rate than the silanized samples. This was attributed to the fast Na^+^ H^+^ ion exchange occurring at the early stage of the glass dissolution, occurring during the conditioning step. The variation in concentration was less pronounced in the case of 13-93B20 due to the lower Na content in the glass composition (Table 1).

It is interesting to note that despite the two glass compositions having almost the same mol % of Ca, the release of this ion happened faster in the case of the borosilicate glass. Indeed, it has been hypothesized that Ca interacts preferentially with the borate network than with the silicate one, which is the least hydrolytically stable.^41,51^ All 13-93B20 BaGs released a higher content of Ca compared to S53P4 BaGs regardless of the treatment, but this amount was significantly higher for the silanized and conditioned 13-93B20. Given the high affinity of Ca and P toward the precipitation of apatite crystals, the high release of Ca, irrespective of the treatment for the glass 13-93B20 is likely to lead to the precipitation of a reactive layer overtime^41,62^

As shown in Figure 8, 13-93B20 released B, K, and Mg, in a similar amount and kinetics regardless of the treatment. This suggested that the borate phase was the most soluble and was not affected by the silica-rich layer formation and Ca–P reactive layer precipitation.

Altogether, these results indicated that (a) the presence of the membrane did not prevent the glass from dissolving, and therefore the ions, beneficial to the cells, were still released to the medium, (b) 13-93B20 glass exhibited a rapidly dissolving borate phase and a stable silicate phase, which in turn promoted membrane stability and higher density of APTES grafting, and (c) 13-93B20 exhibited an ion release profile favorable to the precipitation of a reactive layer.

#### 3.2.3 Membrane Surface Analysis

**Figure 9:**
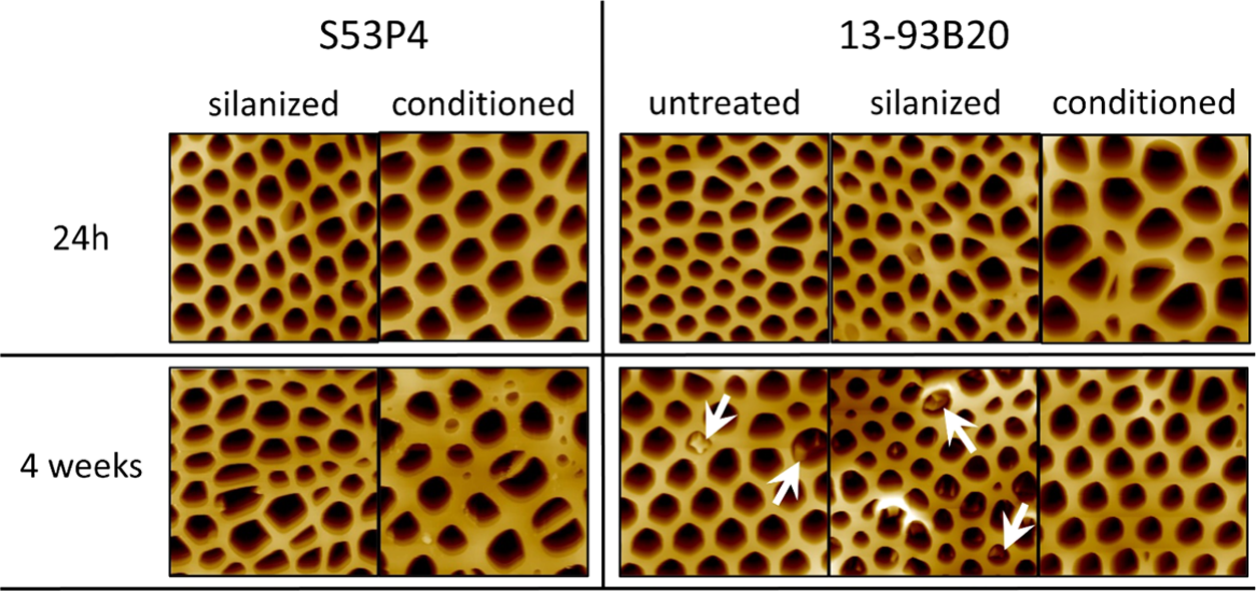
AFM images of the films deposited on the different substrates after incubation in TRIS buffer solution at 37 °C for 24 h and 4 weeks (each image is 30 μm × 30 μm, and each image is from different samples). The white arrows show precipitates.

To assess the surface features of the membrane after immersion, samples incubated in TRIS for 4 weeks were air-dried overnight and imaged by AFM (Figure 9).

The honeycomb structure of the membrane was preserved for at least 4 weeks of immersion in TRIS buffer solution. Images were further processed with Fiji, and the pore size was estimated. Regardless of the incubation time or the substrate, pores were estimated to have a diameter in the 1–5 μm range. The pore size postincubation was similar, within the accuracy of the measurement and the accuracy of the processing, to the sample preincubation.

**Figure 10:**
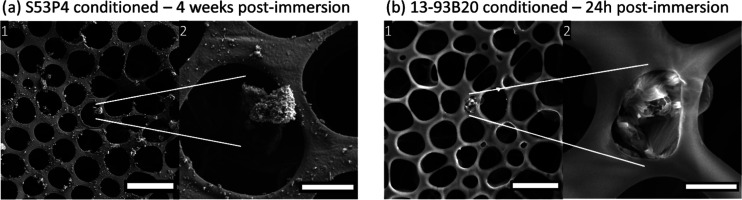
SEM images of the films deposited on (a) conditioned S53P4 or (b) conditioned 13-93B20 incubated in TRIS for 4 weeks and 24 h, respectively (a1 and b1 Scale bar 10 μm. Area of interest a2 and b2 are displayed on the right of the images, Scale bar 2 μm).

To illustrate the precipitation within pores, Figure 10 exhibits the membrane surface of (a) conditioned S53P4 immersed for 4 weeks in TRIS and (b) conditioned 13-93B20 immersed for 24 h in TRIS.

From the SEM images (Figure 10a), one can observe the presence of small nodules at the surface of the membranes deposited on the conditioned S53P4; similar features were also seen at the surface of the silanized S53P4 postimmersion. From Figure 10b, one can see that large aggregates were present within the pores of the membrane. Such aggregates were not visible in the postimmersion of silanized and untreated samples. The EDX analysis revealed a high concentration of Ca and P. Those nodules, both on membrane deposited on S53P4 and 13-93B20, were due to the precipitation of a CaP layer, as expected upon immersion of BaGs.^63^ However, the small size and low density of the nodules did not enable unambiguous EDX analysis.

## 4 Conclusions

In this study, the impact of the bioactive glass surface treatment on the stability of a polymeric membrane deposited using the breath figure method was investigated.

All membranes exhibited a honeycomb-like surface topography, regardless of the BaG composition or the surface modification. The pores of the honeycomb had a diameter ranging from 1 to 5 μm, demonstrating the ability of BaG discs to support the production of a microstructured membrane.

Deposition of a PLDLA membrane on an untreated bioactive glass surface was revealed to yield suboptimal results. Indeed, in dry conditions, membranes demonstrated low resistance to shear, irrespective of the glass composition. Upon immersion, for 4 weeks, all the membranes detached from the S53P4 substrate, while half of them detached from 13-93B20. Therefore, one may conclude that the presence of OH^–^ groups at the material surface was not sufficient to enable strong electrostatic interactions between BaG discs and membranes, leading to early failure of the membrane/BaG disc assembly.

Upon deposition of the membrane on a silanized bioactive glass surface, the presence of amine groups led to a significant enhancement of the membrane adherent properties both in dry and wet conditions. However, it appeared that the improvement was directly linked to the density of the primary amines at the glass surface. Such treatment was found more efficient in the case of 13-93B20 BaG which is assumed to have a faster initial degradation rate. It is believed that the primary amine groups interact, through hydrogen bonds, with PLDLA carbonyl groups.

Finally, deposition of the membrane on conditioned surfaces was revealed to be more effective in reaching a stable BaG disc/membrane interface in dry and wet conditions. The reason for the increased interaction between the BaG disc surface and the membrane appeared to be mainly linked to (1) the precipitation of a reactive layer (CaP) and (2) the subsequent change in topography. Results were significantly better when the membrane was deposited on the 13-93B20 BaG disc than on the S53P4 BaG disc. This was assigned to the thicker and denser reactive layer formed at the surface of this BaG disc compared to the one at the surface of S53P4.

To conclude, this study demonstrated that a PLDLA membrane can be deposited on inorganic surfaces using the breath figure method. With appropriate surface treatment, it was possible to increase the membrane stability. This study also highlighted the capacity of BaGs to maintain a biologically relevant release of ions, even after surface treatment. Results also suggested a potential precipitation of CaP at the membrane surface upon immersion. However, further studies are required to unambiguously identify the composition of the precipitates. The results of this study are promising for the development of new biphasic materials for bone tissue engineering.

## Supporting Information Available

SEM micrograph of the S53P4 substrate after 24 h of immersion in TRIS buffer solution along with the EDX line scan showing the remnant glass, the SiO_2_-rich layer, and the precipitated reactive layer (PDF)

## References

[ref1] Moore W. R.; Graves S. E.; Bain G. I. Synthetic Bone Graft Substitutes. ANZ J. Surg. 2001, 71, 354–361. 10.1046/j.1440-1622.2001.2128.x. 11409021

[ref2] Amini A. R.; Laurencin C. T.; Nukavarapu S. P. Bone Tissue Engineering: Recent Advances and Challenges. Biomed. Eng. 2013, 40, 363–408. 10.1615/CritRevBiomedEng.v40.i5.10. PMC3766369 23339648

[ref3] Wang W.; Yeung K. W. K. Bone Grafts and Biomaterials Substitutes for Bone Defect Repair: A Review. Bioact. Mater. 2017, 2, 224–247. 10.1016/j.bioactmat.2017.05.007. 29744432 PMC5935655

[ref4] Fedarko N. S.; D’Avis P.; Frazier C. R.; Burrill M. J.; Fergusson V.; Tayback M.; Sponseller P. D.; Shapiro J. R. Cell Proliferation of Human Fibroblasts and Osteoblasts in Osteogenesis Imperfecta: Influence of Age. J. Bone Miner. Res. 1995, 10, 1705–1712. 10.1002/jbmr.5650101113. 8592947

[ref5] Dimitriou R.; Mataliotakis G. I.; Calori G. M.; Giannoudis P. V. The Role of Barrier Membranes for Guided Bone Regeneration and Restoration of Large Bone Defects: Current Experimental and Clinical Evidence. BMC Med. 2012, 10, 81 10.1186/1741-7015-10-81. 22834465 PMC3423057

[ref6] Ogiso B.; Hughes F. J.; Melcher A. H.; McCulloch C. A. G. Fibroblasts Inhibit Mineralised Bone Nodule Formation by Rat Bone Marrow Stromal Cells in Vitro. J. Cell. Physiol. 1991, 146, 442–450. 10.1002/jcp.1041460315. 2022698

[ref7] Meinig R. P. Clinical Use of Resorbable Polymeric Membranes in the Treatment of Bone Defects. Orthop. Clin. North Am. 2010, 41, 39–47. 10.1016/j.ocl.2009.07.012. 19931051

[ref8] Kellomäki M.; Puumanen K.; Ashammakhi N.; Waris T.; Paasimaa S.; Törmälä P. Bioabsorbable Laminated Membranes for Guided Bone Regeneration. Technol. Health Care 2002, 10, 165–172. 10.3233/THC-2002-103-402. 12118139

[ref9] Cheng X.; Yang F. More Than Just a Barrier—Challenges in the Development of Guided Bone Regeneration Membranes. Matter 2019, 1, 558–560. 10.1016/j.matt.2019.08.009.

[ref10] Todd V. Scantlebuty. 1982-1992: A Decade of Technology Development for Guided Tissue Regeneration. J. Periodontol. 1993, 64, 1129–1137. 10.1902/jop.1993.64.11s.1129. 8295101

[ref11] Gottlow J. Guided Tissue Regeneration Using Bioresorbable and Non-Resorbable Devices: Initial Healing and Long-Term Results. J. Periodontol. 1993, 64, 1157–1165. 10.1902/jop.1993.64.11s.1157. 8295105

[ref12] Gutta R.; Baker R. A.; Bartolucci A. A.; Louis P. J. Barrier Membranes Used for Ridge Augmentation: Is There an Optimal Pore Size?. J. Oral Maxillofac. Surg. 2009, 67, 1218–1225. 10.1016/j.joms.2008.11.022. 19446207

[ref13] Wessing B.; Lettner S.; Zechner W. Guided Bone Regeneration with Collagen Membranes and Particulate Graft Materials: A Systematic Review and Meta-Analysis. Int. J. Oral Maxillofac. Implants 2018, 33, 87–100. 10.11607/jomi.5461. 28938035

[ref14] Sankar A. R.; Gujjari S. K.; Kulkarni P. K.; Akhila A. R. Development of Biodegradable Silkworm Cocoon Derived Silk Membrane for GTR in the Treatment of Grade II Furcation. Int. J. Res. Pharm. Sci. 2020, 11, 1551–1561. 10.26452/ijrps.v11i2.2033.

[ref15] Reynolds M. A.; Aichelmann-Reidy M. E.; Branch-Mays G. L.; Gunsolley J. C. The Efficacy of Bone Replacement Grafts in the Treatment of Periodontal Osseous Defects. A Systematic Review. Ann. Periodontol. 2003, 8, 227–265. 10.1902/annals.2003.8.1.227. 14971256

[ref16] Yadav V. S.; Narula S. C.; Sharma R. K.; Tewari S.; Yadav R. Clinical Evaluation of Guided Tissue Regeneration Combined with Autogenous Bone or Autogenous Bone Mixed with Bioactive Glass in Intrabony Defects. J. Oral Sci. 2011, 53, 481–488. 10.2334/josnusd.53.481. 22167034

[ref17] Proussaefs P.; Lozada J. The Use of Resorbable Collagen Membrane in Conjunction with Autogenous Bone Graft and Inorganic Bovine Mineral for Buccal/Labial Alveolar Ridge Augmentation: A Pilot Study. J. Prosthet. Dent. 2003, 90, 530–538. 10.1016/S0022-3913(03)00521-3. 14668753

[ref18] Donos N.; Lang N. P.; Karoussis I. K.; Bosshardt D.; Tonetti M.; Kostopoulos L. Effect of GBR in Combination with Deproteinized Bovine Bone Mineral and/or Enamel Matrix Proteins on the Healing of Critical-Size Defects. Clin. Oral Implants Res. 2004, 15, 101–111. 10.1111/j.1600-0501.2004.00986.x. 14731183

[ref19] Calejo M. T.; Ilmarinen T.; Skottman H.; Kellomäki M. Breath Figures in Tissue Engineering and Drug Delivery: State-of-the-Art and Future Perspectives. Acta Biomater. 2018, 66, 44–66. 10.1016/j.actbio.2017.11.043. 29183847

[ref20] Dou Y.; Jin M.; Zhou G.; Shui L. Breath Figure Method for Construction of Honeycomb Films. Membranes 2015, 5, 399–424. 10.3390/membranes5030399. 26343734 PMC4584288

[ref21] Zhang A.; Bai H.; Li L. Breath Figure: A Nature-Inspired Preparation Method for Ordered Porous Films. Chem. Rev. 2015, 115, 9801–9868. 10.1021/acs.chemrev.5b00069. 26284609

[ref22] Wu X. H.; Wu Z. Y.; Su J. C.; Yan Y. G.; Yu B. Q.; Wei J.; Zhao L. M. Nano-Hydroxyapatite Promotes Self-Assembly of Honeycomb Pores in Poly(l-Lactide) Films through Breath-Figure Method and MC3T3-E1 Cell Functions. RSC Adv. 2015, 5, 6607–6616. 10.1039/C4RA13843K.

[ref23] Zhao C.; Pan C.; Sandstedt J.; Fu Y.; Lindahl A.; Liu J. Combination of Positive Charges and Honeycomb Pores to Promote MC3T3-E1 Cell Behaviour. RSC Adv. 2015, 5, 42276–42286. 10.1039/C5RA00756A.

[ref24] Hench L. L. The Story of Bioglass. J Mater. Sci.: Mater. Med. 2006, 17, 967–978. 10.1007/s10856-006-0432-z. 17122907

[ref25] El-Rashidy A. A.; Roether J. A.; Harhaus L.; Kneser U.; Boccaccini A. R. Regenerating Bone with Bioactive Glass Scaffolds: A Review of in Vivo Studies in Bone Defect Models. Acta Biomater. 2017, 62, 1–28. 10.1016/j.actbio.2017.08.030. 28844964

[ref26] Hoppe A.; Güldal N. S.; Boccaccini A. R. A Review of the Biological Response to Ionic Dissolution Products from Bioactive Glasses and Glass-Ceramics. Biomaterials 2011, 32, 2757–2774. 10.1016/j.biomaterials.2011.01.004. 21292319

[ref27] Gorustovich A. A.; Roether J. A.; Boccaccini A. R. Effect of Bioactive Glasses on Angiogenesis: A Review of *In Vitro* and *In Vivo* Evidences. Tissue Eng., Part B 2010, 16, 199–207. 10.1089/ten.teb.2009.0416. 19831556

[ref28] Drago L.; Toscano M.; Bottagisio M. Recent Evidence on Bioactive Glass Antimicrobial and Antibiofilm Activity: A Mini-Review. Materials 2018, 11, 326 10.3390/ma11020326. PMC5849023 29495292

[ref29] Drago L.; Vassena C.; Fenu S.; Vecchi E. D.; Signori V.; Francesco R. D.; Romanò C. L. In Vitro Antibiofilm Activity of Bioactive Glass S53P4. Future Microbiol. 2014, 9, 593–601. 10.2217/fmb.14.20. 24957087

[ref30] Sayed Abdelgeliel A.; Ferraris S.; Cochis A.; Vitalini S.; Iriti M.; Mohammed H.; Kumar A.; Cazzola M.; Salem W. M.; Verné E.; Spriano S.; Rimondini L. Surface Functionalization of Bioactive Glasses with Polyphenols from Padina Pavonica Algae and In Situ Reduction of Silver Ions: Physico-Chemical Characterization and Biological Response. Coatings 2019, 9, 394 10.3390/coatings9060394.

[ref31] Vernè E.; Ferraris S.; Vitale-Brovarone C.; Cochis A.; Rimondini L. Bioactive Glass Functionalized with Alkaline Phosphatase Stimulates Bone Extracellular Matrix Deposition and Calcification in Vitro. Appl. Surf. Sci. 2014, 313, 372–381. 10.1016/j.apsusc.2014.06.001.

[ref32] Philippart A.; Boccaccini A. R.; Fleck C.; Schubert D. W.; Roether J. A. Toughening and Functionalization of Bioactive Ceramic and Glass Bone Scaffolds by Biopolymer Coatings and Infiltration: A Review of the Last 5 Years. Expert Rev. Med. Devices 2015, 12, 93–111. 10.1586/17434440.2015.958075. 25331196

[ref33] Stanić V. Variation in Properties of Bioactive Glasses After Surface Modification. In Clinical Applications of Biomaterials; Kaur G., Ed.; Springer International Publishing: Cham, 2017; pp 35–63.

[ref34] Calejo M. T.; Ilmarinen T.; Jongprasitkul H.; Skottman H.; Kellomäki M. Honeycomb Porous Films as Permeable Scaffold Materials for Human Embryonic Stem Cell-Derived Retinal Pigment Epithelium: Porous Films as Scaffolds for HESC-RPE. J. Biomed. Mater. Res. 2016, 104, 1646–1656. 10.1002/jbm.a.35690. 26914698

[ref35] Hupa L. Composition-Property Relations of Bioactive Silicate Glasses. In Bioactive Glasses; Elsevier, 2018; pp 1–35.

[ref36] Anderson Ö.H.; Karlson K. H.; Liu G.; Niemi L. In Vivo Behavior of Glasses in the SiO2-NaO2-CaO-P2O5-Al2O3-B2O3 System. J. Mater. Sci.: Mater. Med. 1990, 1, 219–227. 10.1007/BF00701080.

[ref37] Houaoui A.; Lyyra I.; Agniel R.; Pauthe E.; Massera J.; Boissière M. Dissolution, Bioactivity and Osteogenic Properties of Composites Based on Polymer and Silicate or Borosilicate Bioactive Glass. Mater. Sci. Eng., C 2020, 107, 110340 10.1016/j.msec.2019.110340. 31761244

[ref38] Massera J.; Mishra A.; Guastella S.; Ferraris S.; Verné E. Surface Functionalization of Phosphate-Based Bioactive Glasses with 3-Aminopropyltriethoxysilane (APTS). Biomed. Glasses 2016, 2, 51–62. 10.1515/bglass-2016-0007.

[ref39] Luxbacher T. The ZETA Guide: Principles of the Streaming Potential Technique; Anton Paar GmbH: Graz, Austria, 2014.

[ref40] Lin M. R.; Ritter J. E.; Rosenfeld L.; Lardner T. J. Measuring the Interfacial Shear Strength of Thin Polymer Coatings on Glass. J. Mater. Res. 1990, 5, 1110–1117. 10.1557/JMR.1990.1110.

[ref41] Ferraris S.; Nommeots-Nomm A.; Spriano S.; Vernè E.; Massera J. Surface Reactivity and Silanization Ability of Borosilicate and Mg-Sr-Based Bioactive Glasses. Appl. Surf. Sci. 2019, 475, 43–55. 10.1016/j.apsusc.2018.12.218.

[ref42] Ferraris S.; Yamaguchi S.; Barbani N.; Cazzola M.; Cristallini C.; Miola M.; Vernè E.; Spriano S. Bioactive Materials: In Vitro Investigation of Different Mechanisms of Hydroxyapatite Precipitation. Acta Biomater. 2020, 102, 468–480. 10.1016/j.actbio.2019.11.024. 31734414

[ref43] Lu H. H.; Pollack S. R.; Ducheyne P. 45S5 Bioactive glass surface charge variations and the formation of a surface calcium phosphate layer in a solution containing fibronectin. J. Biomed. Mater. Res. 2001, 54, 454–461. 10.1002/1097-4636(20010305)54:33.0.CO;2-H. 11189054

[ref44] Lu H. H.; Pollack S. R.; Ducheyne P. Temporal zeta potential variations of 45S5 bioactive glass immersed in an electrolyte solution. J. Biomed. Mater. Res. 1999, 51, 80–87. 10.1002/(SICI)1097-4636(200007)51:13.0.CO;2-6. 10813748

[ref45] Doostmohammadi A.; Monshi A.; Salehi R.; Fathi M. H.; Karbasi S.; Pieles U.; Daniels A. U. Preparation, Chemistry and Physical Properties of Bone-Derived Hydroxyapatite Particles Having a Negative Zeta Potential. Mater. Chem. Phys. 2012, 132, 446–452. 10.1016/j.matchemphys.2011.11.051.

[ref46] Brown R. F.; Rahaman M. N.; Dwilewicz A. B.; Huang W.; Day D. E.; Li Y.; Bal B. S. Effect of Borate Glass Composition on Its Conversion to Hydroxyapatite and on the Proliferation of MC3T3-E1 Cells. J. Biomed. Mater. Res., Part A 2009, 88, 392–400. 10.1002/jbm.a.31679. 18306284

[ref47] Fu Q.; Rahaman M. N.; Fu H.; Liu X. Silicate, Borosilicate, and Borate Bioactive Glass Scaffolds with Controllable Degradation Rate for Bone Tissue Engineering Applications. I. Preparation and in Vitro Degradation. J. Biomed. Mater. Res., Part A 2010, 95, 164–171. 10.1002/jbm.a.32824. 20544804

[ref48] Huang W.; Day D. E.; Kittiratanapiboon K.; Rahaman M. N. Kinetics and Mechanisms of the Conversion of Silicate (45S5), Borate, and Borosilicate Glasses to Hydroxyapatite in Dilute Phosphate Solutions. J. Mater. Sci.: Mater. Med. 2006, 17, 583–596. 10.1007/s10856-006-9220-z. 16770542

[ref49] Varila L.; Fagerlund S.; Lehtonen T.; Tuominen J.; Hupa L. Surface Reactions of Bioactive Glasses in Buffered Solutions. J. Eur. Ceram. Soc. 2012, 32, 2757–2763. 10.1016/j.jeurceramsoc.2012.01.025.

[ref50] Massera J.; Hupa L. Influence of SrO Substitution for CaO on the Properties of Bioactive Glass S53P4. J. Mater. Sci.: Mater. Med. 2014, 25, 657–668. 10.1007/s10856-013-5120-1. 24338267

[ref51] Tainio J. M.; Salazar D. A. A.; Nommeots-Nomm A.; Roiland C.; Bureau B.; Neuville D. R.; Brauer D. S.; Massera J. Structure and in Vitro Dissolution of Mg and Sr Containing Borosilicate Bioactive Glasses for Bone Tissue Engineering. J. Non-Cryst. Solids 2020, 533, 119893 10.1016/j.jnoncrysol.2020.119893.

[ref52] Balasubramanian P.; Büttner T.; Miguez Pacheco V.; Boccaccini A. R. Boron-Containing Bioactive Glasses in Bone and Soft Tissue Engineering. J. Eur. Ceram. Soc. 2018, 38, 855–869. 10.1016/j.jeurceramsoc.2017.11.001.

[ref53] Schuhladen K.; Wang X.; Hupa L.; Boccaccini A. R. Dissolution of Borate and Borosilicate Bioactive Glasses and the Influence of Ion (Zn, Cu) Doping in Different Solutions. J. Non-Cryst. Solids 2018, 502, 22–34. 10.1016/j.jnoncrysol.2018.08.037.

[ref54] Bormashenko E. Breath-Figure Self-Assembly, a Versatile Method of Manufacturing Membranes and Porous Structures: Physical, Chemical and Technological Aspects. Membranes 2017, 7, 45 10.3390/membranes7030045. PMC5618130 28813026

[ref55] Meroni D.; Lo Presti L.; Di Liberto G.; Ceotto M.; Acres R. G.; Prince K. C.; Bellani R.; Soliveri G.; Ardizzone S. A Close Look at the Structure of the TiO_2_ -APTES Interface in Hybrid Nanomaterials and Its Degradation Pathway: An Experimental and Theoretical Study. J. Phys. Chem. C 2017, 121, 430–440. 10.1021/acs.jpcc.6b10720. PMC5295244 28191270

[ref56] Lai Y.; Zeng Y.; Tang X.; Zhang H.; Han J.; Su H. Structural Investigation of Calcium Borosilicate Glasses with Varying Si/Ca Ratios by Infrared and Raman Spectroscopy. RSC Adv. 2016, 6, 93722–93728. 10.1039/C6RA20969F.

[ref57] Zhou S.; Zheng X.; Yu X.; Wang J.; Weng J.; Li X.; Feng B.; Yin M. Hydrogen Bonding Interaction of Poly(d,l-Lactide)/Hydroxyapatite Nanocomposites. Chem. Mater. 2007, 19, 247–253. 10.1021/cm0619398.

[ref58] Brink M.; Turunen T.; Happonen R.-P.; Yli-Urpo A. Compositional dependence of bioactivity of glasses in the system Na2O-K2O-MgO-CaO-B2O3-P2O5-SiO2. J. Biomed. Mater. Res. 1997, 37, 114–121. 10.1002/(SICI)1097-4636(199710)37:13.0.CO;2-G. 9335356

[ref59] Yu Y.; Stevensson B.; Edén M. Medium-Range Structural Organization of Phosphorus-Bearing Borosilicate Glasses Revealed by Advanced Solid-State NMR Experiments and MD Simulations: Consequences of B/Si Substitutions. J. Phys. Chem. B 2017, 121, 9737–9752. 10.1021/acs.jpcb.7b06654. 28876931

[ref60] Mishra A.; Rocherullé J.; Massera J. Ag-Doped Phosphate Bioactive Glasses: Thermal, Structural and in-Vitro Dissolution Properties. Biomed. Glasses 2016, 2, 38–48. 10.1515/bglass-2016-0005.

[ref61] Hench L. L.; Andersson O. Bioactive glasses: An Introduction to bioceramics. Adv. Ser. Ceram. 2011, 41–62. 10.1142/9789814317351_0003.

[ref62] Bohner M.; Lemaitre J. Can Bioactivity Be Tested in Vitro with SBF Solution?. Biomaterials 2009, 30, 2175–2179. 10.1016/j.biomaterials.2009.01.008. 19176246

[ref63] Hench L. L. Chronology of Bioactive Glass Development and Clinical Applications. New J. Glass Ceram. 2013, 3, 67–73. 10.4236/njgc.2013.32011.

