# Supplementary material for: Polymer-Based Honeycomb Films on Bioactive Glass: Toward a Biphasic Material for Bone Tissue Engineering Applications
Source: ACS Appl Mater Interfaces. 2021 Jun 15;13(25):29984–95. doi: 10.1021/acsami.1c03759 (PMC8289249; doi:10.1021/acsami.1c03759)
Supplement: Supplementary file 1 — am1c03759_si_001.pdf [file am1c03759_si_001.pdf]

Polymer-based honeycomb films on bioactive glass: toward a biphasic material for bone tissue engineering applications

A.Deraine [a,b] , M. T. Rebelo Calejo[b], R.Agniel [a], M.Kellomäki [b] , E.Pauthe [a] , M.Boissière [a] , **J.Massera** [b*]

[a] ERRMECe, Equipe de Recherche sur les Relations Matrice Extracellulaire-Cellules (EA1391), Université de Cergy-Pontoise, Maison Internationale de la Recherche (MIR), rue Descartes, 95001 Neuville sur Oise Cedex, France

[b] Laboratory of Biomaterials and Tissue Engineering, Faculty of Medicine and Health Technology, Tampere University, Korkeakoulunkatu 3, 33720 Tampere, Finland

[*] **corresponding author, email: jonathan.massera@tuni.fi**

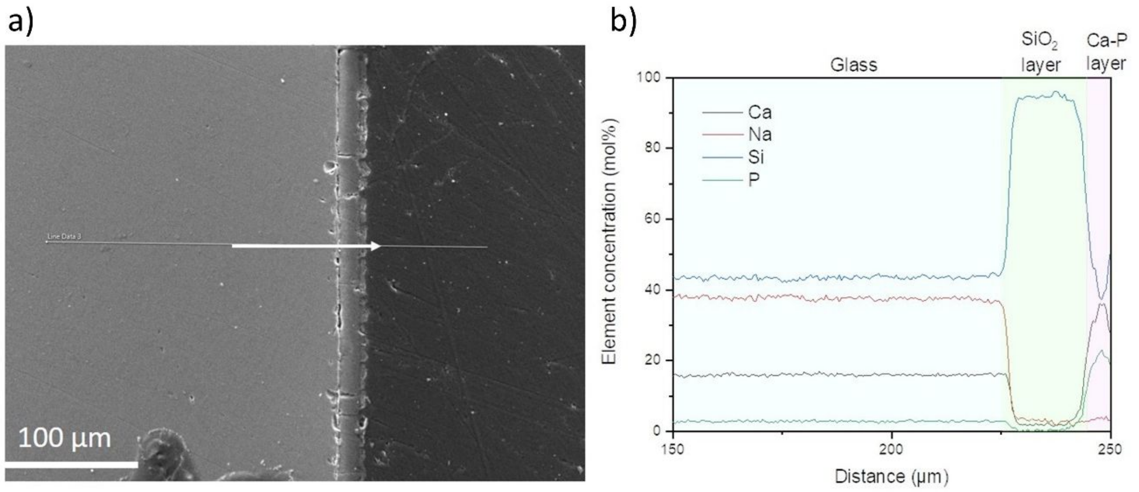

Figure S1. a) SEM micrograph of S53P4 after 24h immersion in TRIS buffer solution, b) EDX line scan. The EDX line scan shows the glass, SiO_2 layer, CaP layer region across the white arrow (on a).
